# Smart Dosing, Better Outcomes: An Electronic Medical Record Intervention for Cancer Pain in the Emergency Department

**DOI:** 10.1016/j.acepjo.2026.100449

**Published:** 2026-06-25

**Authors:** Christopher J. Coyne, Ryan Miller, Kelly Dong, Anthony S. Tadros

**Affiliations:** 1 Department of Emergency Medicine, University of California San Diego, San Diego, California, USA; 2 Western University of Health Sciences, Pomona, California, USA; 3 Division of Interventional Radiology, Department of Radiology, University of California San Diego, San Diego, California, USA

**Keywords:** oncology, cancer, pain, palliative care, decision-support, analgesia, opioids

## Abstract

**Objectives:**

We hypothesized that an electronic medical record (EMR)-based best practice advisory (BPA), offering individualized opioid dosing for cancer patients with high opioid tolerance, would improve pain control and reduce hospital admissions in the emergency department (ED).

**Methods:**

We conducted a retrospective cohort study from May 2020 to May 2024 across 2 academic EDs affiliated with a National Comprehensive Cancer Network cancer center. Adult patients with active cancer, a morphine equivalent daily dose (MEDD) ≥100, and an ED opioid order were eligible. The BPA calculated MEDD-based opioid dosing recommendations and provided safety measures. Patients were categorized as BPA+ (recommendation followed) or BPA− (recommendation ignored). The primary outcome was hospital admission. Secondary outcomes included ED length of stay and change in pain scores.

**Results:**

Among 1193 patient encounters, 719 (60.3%) received BPA-directed therapy. BPA+ was associated with a lower odds ratio (OR) of admission compared to BPA− (OR, 0.67; 95% CI, 0.49-0.80). The BPA was associated with decreased admission rates in patients with an Eastern Cooperative Oncology Group performance status of 4 (54% vs 80% admitted) and stage 4 cancer (57% vs 68% admitted). BPA+ was also associated with greater median pain improvement (−2 vs −1) and shorter ED stays (8.2 vs 8.9 hours). Among patients receiving multiple opioid doses, greater BPA adherence was associated with sustained pain reduction, while BPA nonadherence showed diminished response.

**Conclusion:**

An EMR-integrated BPA for opioid-tolerant cancer patients was associated with improved ED pain management and reduced admissions. These findings support broader use of EMR-based tools to promote guideline-concordant, personalized care for cancer-related pain.

**The Bottom Line**

Cancer pain in the emergency department is common and difficult to manage, particularly in patients already taking high-dose opioids, where individualized dosing can be challenging in a time-sensitive setting. In this study of 1193 emergency department visits at 2 hospitals, an electronic medical record tool provided personalized opioid dosing recommendations based on each patient’s home regimen. Approximately 60% of patients received tool-guided care, which was associated with lower hospital admission rates (adjusted odds ratio, 0.67), greater pain improvement (median reduction 2 vs 1 points), and shorter emergency department stays (8.2 vs 8.9 hours), with no adverse events. This matters because real-time decision support can help standardize care and improve outcomes for cancer patients presented to the ED with pain.

## 1 Introduction

### 1.1 Background

Effective pain control is a cornerstone of quality cancer care; yet for many patients, especially those presenting to the emergency department (ED), this fundamental need remains unmet. Up to 86% of patients with advanced cancer experience pain, and ∼40% of cancer-related ED visits involve pain as a chief symptom.^1^^,^^2^ Despite this, studies have shown that nearly 1 in 3 patients with cancer-related pain receive no analgesia during their ED visit, and those who do often wait over 90 minutes for initial pain management—well beyond the standards recommended by national pain guidelines.^3^^,^^4^

### 1.2 Importance

The ED represents a critical touchpoint for patients experiencing uncontrolled cancer pain, especially during off-hours when outpatient oncology or palliative care support is unavailable. However, pain management in this setting is frequently compromised by fragmented communication, lack of oncology-specific clinical context, variability in provider comfort with opioids, and the residual effects of the opioid epidemic.^5^ Emergency physicians (EP) must often make complex pain management decisions without access to patients’ cancer diagnoses, treatment history, or prior pain control regimens. Timely access to oncology notes or palliative care consults are persistent issues in many EDs, contributing to undertreatment and unnecessary admissions.^6^

Electronic medical record (EMR) systems offer a promising avenue to address these systemic deficiencies. Integration of oncology-specific pain management protocols, smart phrases that autopopulate opioid titration plans, and clinical decision support tools can help standardize care and reduce treatment delays.^7^^,^^8^ Additionally, EMR-based pain history snapshots, such as flags for chronic opioid use, prior pain scores, or recent palliative care involvement, can guide more appropriate prescribing and avoid redundant workups.^9^^,^^10^ Implementation of EMR alerts or care pathways have already shown success in other time-sensitive conditions such as sepsis and stroke; similar models could be adapted for cancer pain.^11^^,^^12^

Cancer patients on chronic opioids often develop tolerance, requiring higher doses for effective pain relief. In the ED, however, they are frequently undertreated due to lack of access to outpatient opioid histories, concerns about oversedation, and stigma around high-dose opioids.^5^ Standard dosing may be insufficient for these patients, leading to inadequate pain control and avoidable admissions. Tailored approaches are needed to address this gap in acute cancer pain management.

### 1.3 Goals of This Investigation

We designed and EMR-based tool that provides individualized opioid dosing regiments for cancer patients presenting to the ED with pain. This study evaluates the effectiveness of this tool in providing appropriate dosing, managing pain scores, and decreasing unnecessary admissions.

## 2 Methods

### 2.1 Study Design and Setting

This is a retrospective cohort study conducted between May 2020 and May 2024 at 2 academic hospitals, one located in an urban environment and the other in a suburban environment. Both hospitals are affiliated with a National Comprehensive Cancer Network (NCCN)-accredited cancer center, and both hospitals are located in Southern California. This study was approved by our local institutional review board. STROBE methods were used to assure the accuracy and completeness of data reporting (Supplementary Appendix 1).^13^

### 2.2 Selection of Participants

All adult patients 18 years of age or older, who were prescribed opioids with a calculated morphine equivalent daily dose (MEDD) of 100 or greater, who presented to a study ED and carried a diagnosis of active cancer were included in the study. Active cancer was defined as a diagnosis of cancer with ongoing treatment (eg, chemotherapy, immunotherapy, radiation therapy, and surgical therapy), ongoing symptoms relating to cancer, or a known cancer recurrence.^3^^,^^4^^,^^14^ Patients were excluded if they were found to have nonmelanomatous skin cancer or carcinoma in situ. We included patients consecutively during our study enrollment period. Data were collected prospectively and analyzed retrospectively.

### 2.3 Interventions

During our study period, all patients with a documented cancer, with at least 1 documented opioid prescription that resulted in an MEDD of 100 or greater, whose EP ordered an oral, intravenous or intramuscular opioid during their ED encounter would receive a best practice advisory (BPA) in their chart (Supplementary Appendixes 2 and 3). The EMR software would automatically calculate the patient’s MEDD and provide a personalized dosing recommendation for the opioid ordered by the EP. This BPA would also provide a range of potential new opioid dosages for the EP to order (accounting for each patients’ home opioid usage and potential tolerance), as well as safety measures such as continuous pulse oximetry, continuous capnography, as well as naloxone (as needed). If the BPA was triggered and the EP used the dosing recommendation or increased their dosage, this was considered BPA+ (intervention). If the BPA was triggered and the EP did not use the dosing recommendation or increase their dose, this was considered BPA− (control). All EPs were required to input their original intended dose prior to accepting or declining the BPA recommendations, to gauge original intent and subsequent decision making.

### 2.4 Measurements

Demographic data were collected for all patients, including age, biological sex, and race/ethnicity. Biological sex was categorized as male or female as documented in the EMR. Race/ethnicity was abstracted from the medical record and analyzed using mutually exclusive categories as recorded, including White, Latinx, Black, mixed race, Asian/Pacific Islander, and Indigenous. Cancer-specific variables included cancer type, cancer stage, and Eastern Cooperative Oncology Group (ECOG) performance status. Cancer type was categorized by primary malignancy site or disease group. Cancer stage was analyzed categorically, with particular attention to stage IV disease given its clinical relevance to pain burden, disposition decisions, and prognosis. ECOG performance status was treated as an ordinal categorical variable ranging from 0 to 4.

Additional categorical variables included code status, prior palliative care involvement, prior hospice involvement, pain-related chief symptom, ED diagnosis, disposition, and mortality. Code status was categorized as full code vs any limitation in resuscitative preferences. Prior palliative care and hospice involvement were coded as present or absent based on documentation before or during the index encounter. Disposition, the primary outcome, was categorized as hospital admission vs discharge from the ED. BPA utilization, the primary exposure, was categorized as BPA+ if the emergency physician used the BPA-recommended opioid dose or increased the originally intended dose after the BPA was triggered, and BPA− if the BPA recommendation was not used and the originally intended dose was not increased. Safety outcomes, including hypoxia, hypotension, and naloxone administration, were coded as binary variables indicating the presence or absence of each event during the ED encounter. Categorical variables were summarized using frequencies and percentages and compared using χ^2^ testing when appropriate.

### 2.5 Outcomes

Our primary outcome measurement was disposition (admit vs discharge), with our primary exposure variable being BPA utilization (BPA+ vs BPA−). Our secondary outcomes included change in pain scores (final pain score minus initial pain score) and ED length of stay (time from patient arrival to disposition from the ED).

### 2.6 Analysis

We conducted a sample size calculation to detect a 10% reduction in overall admission rate, with an enrollment ratio of 0.6, an α of 0.05, and a power of 80%. This calculation resulted in a necessary sample size of at least 827 participants.

For our primary outcome, we used multivariable logistic regression to assess the association between BPA utilization and hospital admission, adjusting for potential confounders and effect modifiers. Model fit was assessed using standard goodness-of-fit measures for logistic regression, including evaluation of calibration and discrimination. Given the multicenter design, site of care was considered as a potential source of confounding. Models were evaluated for site-level effects, and inclusion of site as a covariate did not meaningfully change the effect estimates; therefore, it was not retained in the final model. Covariates were selected a priori based on clinical relevance and prior literature rather than automated statistical selection. The prespecified covariates included age, biological sex, ECOG performance status, cancer stage, cancer type, and prior palliative care involvement. These variables were chosen because they represent patient demographics, functional status, disease severity, malignancy characteristics, and prior supportive care involvement, each of which may plausibly influence both BPA utilization and hospital admission. We explored potential effect modification by ECOG performance status and cancer stage through stratified analyses, given their clinical relevance to pain management and disposition decisions. Formal interaction terms were not retained in the final model due to lack of statistical significance. For simple comparisons, we used Welch *T* test to compare means of normally distributed data, χ^2^ analysis to compare nonnormally distributed categorical data, as well as Mann–Whitney *U* test to compare distributions/medians, when appropriate. Missing data were minimal and handled using complete case analysis, with only encounters containing complete data for variables included in the model retained. All data were analyzed using SPSS version 29 (IBM).

## 3 Results

### 3.1 Overall Cohort Characteristics

**Figure 1:**
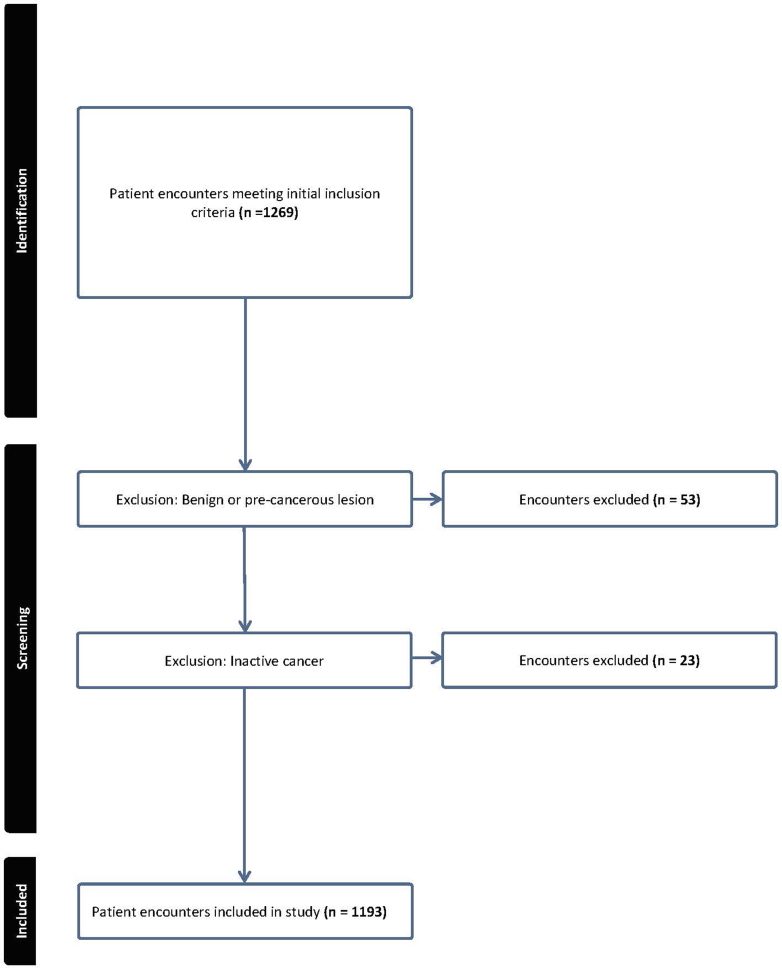
Flowchart illustrating patient selection.

During our study period, we evaluated 1269 unique patient encounters that met inclusion criteria. We then excluded 53 patients who were found to have nonmelanomatous skin cancer or carcinoma in situ and 23 patients who were found to be in cancer remission, for a total of 1193 patient encounters included in the final analysis (Fig 1).

The overall cohort had a median age of 55 years, was just over half women (600, 50.3%), and was predominantly White (683,57.3%), followed by Latinx (204, 17.1%), Black (141, 11.8%), and mixed race (99, 8.3%) (Table 1).

**Table 1:** Characteristics of study population including demographics, cancer characteristics, and disposition.

Characteristic	BPA used, 719 (60.3)	BPA not used, 474 (39.7)	Total, 1193 (100)
Sex (female)	372 (51.7)	228 (48.1)	600 (50.3)
Age (y)	53	57.5	55
Race/ethnicity			
White	401 (55.8)	282 (59.5)	683 (57.3)
Latinx	126 (17.5)	78 (16.5)	204 (17.1)
Black	87 (12.1)	54 (11.4)	141 (11.8)
Mixed race	56 (7.9)	43 (8.9)	99 (8.3)
Asian/Pacific Islander	37 (5)	14 (3.2)	51 (4.3)
Indigenous	12 (1.7)	3 (.6)	15 (1.3)
Pain-related chief symptom	467 (65.0)	228 (48.1)	695 (58.3)
ECOG			
0	27 (3.8)	9 (1.9)	36 (3)
1	132 (18.4)	81 (17.1)	213 (17.9)
2	248 (34.6)	178 (37.3)	426 (35.7)
3	201 (27.8)	131 (27.8)	332 (27.8)
4	111 (15.4)	75 (15.8)	186 (15.6)
Cancer stage			
1	25 (3.4)	11 (2.4)	36 (3)
2	40 (5.7)	28 (5.7)	68 (5.7)
3	113 (15.8)	46 (9.6)	159 (13.3)
4	418 (58.1)	290 (61.4)	708 (59.3)
Unstageable	123 (17.0)	99 (20.9)	222 (18.6)
Code status			
Full code	624 (86.7)	405 (85.4)	1029 (86.3)
DNR/DNI + full care	70 (9.8)	37 (7.6)	107 (9)
DNR/DNI + comfort care	19 (2.6)	26 (5.7)	45 (3.8)
Other	6 (.8)	6 (1.3)	12 (1)
Palliative care (yes)	421 (58.5)	290 (61.4)	711 (59.6)
Hospice (yes)	19 (2.5)	29 (6.3)	48 (4)
Pain-related ED diagnosis (yes)	255 (35.3)	131 (27.6)	386 (32.4)
ED LOS (h)	8.2	8.9	8.5
ED disposition (admit)	374 (52)	303 (63.9)	677 (56.7)
ED death	0	2 (.4)	2 (.2)
Admit LOS (d)	5	6	6
Admit death	31 (4.3)	14 (2.9)	45 (3.8)
Pain-related hospital diagnosis	91 (21.5)	62 (20.6)	153 (21.1)

BPA used refers to opioid dosing was adjusted as recommended.

BPA, best practice advisory; DNI, do not intubate; DNR, do not resuscitate; ECOG, Eastern Cooperative Oncology Group; ED, emergency department; LOS, length of stay.

Overall cancer characteristics describe a diverse cohort, with colorectal cancer being the most prevalent in our study (277, 23%), followed by gastrointestinal (153, 12.8%), reproductive female (111, 9.3%), and breast (102, 8.5%) (Table 2). The most common ECOG performance status was 2, which describes a patient with work limitations but is able to care for oneself. Contrastingly, the disease burden in our cohort was quite high, with ∼60% of the patients presenting with stage 4 cancer. A similar amount had previously been seen by palliative care on at least 1 occasion (711, 59.6%), while only a small fraction had been seen by hospice (48, 4%). Finally, despite the level of advanced disease in our cohort, the vast majority were considered full code (1029, 86.3%).

**Table 2:** Patient characteristics, pain outcomes, and disposition by cancer type.

Cancer type (n)	Median stage	Initial pain score	Final pain score	δ pain score	Admission rate (%)	Admitted for pain (% of admissions)
Colorectal (277)	4	8	7	−2	68.5	27
Gastrointestinal (153)	4	8	7	−2	64.7	30
Reproductive Female (111)	3	8	7	0	56.8	19
Breast (102)	4	7.5	6	−3	54.3	10.5
Urinary (100)	4	8	7	−1	75.8	28
Leukemia/Lymphoma (89)	NA	7	6	0	63.3	21
Head and Neck (88)	4	8	7	−1	46.7	29
Lung (64)	4	8	5	−3	66	15
Reproductive Male (63)	4	7	6	−1	52.4	27
Sarcoma (35)	4	8	7	−1	41.7	60
Melanoma (35)	4	8	8	0	77.1	20
Endocrine (34)	2	10	8	−2	0	0
Neuroendocrine (17)	4	5.5	5	1	66.7	0
CNS (16)	3	8	0	−6	40	0
Unspecified (9)	NA	10	8	0	100	33
Total (1193)	4	8	7	−1	56.7	20.8

Values are presented as medians unless otherwise specified. Admission rate represents the proportion of encounters resulting in hospital admission. Admissions for pain reflects the proportion of admitted patients for whom pain was the primary reason for admission.

Regarding overall pain characteristics, our results reflect the refractory nature of cancer pain. The median initial pain score among our cohort was 8, with a minimal improvement upon disposition, with a median final pain score of 7 and a median change in pain score of −1 (Table 2). Certain cancer types appeared to be more refractory to analgesia than others, with hematologic malignancies and metastatic melanoma having no improvement in median pain, and neuroendocrine tumors demonstrating an increase in pain at time of disposition with a median change in pain score of +1. Although the overall percent of admissions for pain control was 20.8% in our cohort, the fraction of admissions for pain control was quite high among certain cancer types including sarcoma (60%), gastrointestinal (30%), and head and neck (29%).

Operational metrics among our cohort reveal an overall high admission rate (677, 56.7%). The median ED length of stay was ∼8.5 hours, while the median hospital length of stay for admitted patients was ∼6 days. Overall, the mortality in our cohort was low despite the high burden of advanced cancer, with 2 ED deaths and 45 in-hospital deaths during the index admission.

### 3.2 Primary Outcome—Hospital Admission

Among our final cohort, nearly two-thirds of patients received BPA-directed therapy during their ED encounter (n = 719, 60.3%). The group of patients who received BPA-directed therapy (BPA+) were similar in nearly all metrics to those whose providers chose not to use BPA directed therapy (BPA−), although the BPA+ group had a higher percentage of patients with a pain-related chief symptom (Table 1).

**Figure 2:**
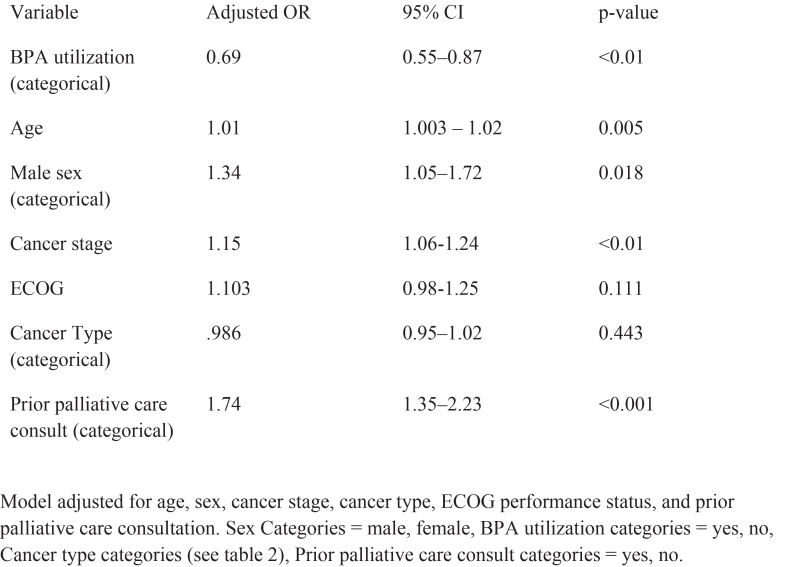
Multivariable logistic regression model for hospital admission.

After adjusting for age, sex, ECOG, stage, and cancer type, the BPA+ was associated with a lower odds ratio (OR) of admission compared with BPA− (adjusted OR, 0.67; 95% CI, 0.49-0.80). Other variables were included in the model as adjustment covariates and were not interpreted as primary effects (Fig 2). Although ECOG score was not independently associated with admission, when stratified, the effect of the BPA on OR of admission for patients with ECOG 4 was significant, with 80% of BPA− patients being admitted, vs 54% of BPA+ patients (OR, 0.29; 95% CI, 0.15-0.58). Similarly, when cancer stage is stratified, the effect of the BPA on OR of admission for patients with stage 4 cancer was significant, with 68% of BPA− patients being admitted, vs 57% of BPA+ patients (OR, 0.63; 95% CI, 0.4609-0.863).

### 3.3 Secondary Outcomes

**Figure 3:**
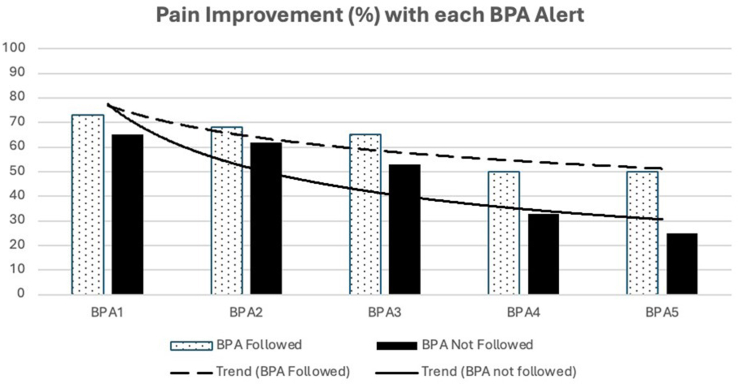
Trend in median pain response (% improvement) after each BPA alert and subsequent analgesic administration based on BPA followed versus BPA not followed. BPA, best practice advisory.

Median ED length of stay was lower in the BPA group (8.2 hours; IQR, 5.5-11.9 hours) compared with the non-BPA group (8.9 hours; IQR, 6.3-13.3; *P* = .004). Similarly, there was a modest, although statistically significant improvement in pain reduction between the BPA+ group vs the BPA− group. Median pain reduction was −2 (IQR, −4 to 0) in the BPA group compared with −1 (IQR, −3 to 0) in the non-BPA group (*P* < .001). The mean difference in pain reduction was −0.73 (95% CI, −1.14 to −0.32). When we evaluated patients that received multiple opioid administrations, and therefore multiple BPAs, we found that for those patients whose providers continued to follow BPA directed dosing, there was a more sustained analgesic effect. This was in contrast to those who did not follow the BPA, whose patients experienced diminished returns with each subsequent opioid administration (Fig 3).

Importantly, there were no adverse outcomes over the course of this study. Specifically, there were no episodes of hypoxia or hypotension. Additionally, there was no need for opioid reversal during any of the patient encounters.

## 4 Limitations

This study has several limitations. It was conducted at 2 academic centers affiliated with an NCCN cancer center, which may limit generalizability to community or nonacademic EDs with fewer resources or different EMR capabilities. Additionally, while data were collected prospectively, the analysis was retrospective in nature, which may introduce bias due to unmeasured confounding factors. Although we adjusted for key variables such as ECOG performance status, cancer stage, and prior palliative care involvement, residual confounding is possible.

The decision to follow or not follow the BPA was left to provider discretion, which may have introduced selection bias. Providers more comfortable with aggressive pain management or more familiar with cancer care may have been more likely to follow BPA recommendations, potentially skewing outcomes. We did not collect information on why the BPA was not used, but this is an area of future investigation. Furthermore, provider familiarity with EMR-based decision support may have influenced BPA uptake. To our knowledge, there were no prior opioid-dosing or cancer pain BPAs in our health system that would have specifically influenced provider willingness to accept these recommendations. However, we did not measure individual provider experience with BPAs or prior practice environments, and this may represent an unmeasured source of variation.

Pain assessment is inherently subjective, and while numeric pain scores are widely used, they may not fully capture the complexity or variability of cancer-related pain experiences across patient populations. We did not assess postdischarge outcomes such as long-term symptom control, functional recovery, or patient satisfaction. As a result, while BPA use was associated with reduced admission rates and improved ED analgesia, it remains unclear whether these benefits translate to sustained improvements after discharge. Future studies should incorporate longitudinal follow-up and patient-reported outcomes to more comprehensively assess the impact of EMR-based interventions in cancer pain management.

Finally, due to the observational design, the findings of this study should be interpreted as associations rather than causal effects. Although BPA-guided care was associated with lower OR of admission, improved pain reduction, and shorter ED length of stay, we cannot determine whether BPA utilization directly caused these outcomes. Provider discretion in accepting or declining BPA recommendations may have introduced selection bias, and residual confounding remains possible despite adjustment for clinically relevant covariates.

## 5 Discussion

Our findings highlight both the high symptom burden experienced by patients with cancer in the ED and the potential of EMR-based interventions to improve care. In this large, diverse cohort of 1,193 patient encounters, pain was a dominant feature, with a median initial pain score of 8 and a median pain improvement of only −1, underscoring the refractory nature of cancer-related pain in the acute setting. With a high prevalence of advanced disease—nearly 60% of patients had Stage 4 cancer and nearly 60% had seen palliative care—pain control remained inadequate for many, with some subgroups (e.g., hematologic malignancies, metastatic melanoma, neuroendocrine tumors) showing no improvement or worsening pain during their ED stay.

The opioid epidemic has had far-reaching consequences on acute pain management, including for patients with cancer in the ED.^15^^,^^16^ While efforts to curb opioid misuse have been necessary, they have also led to increased scrutiny and hesitation around opioid prescribing—even in populations for whom opioids are medically appropriate and essential.^17^ EPs, operating in a high-stakes environment and often without complete oncology records, may feel pressure to under-prescribe or delay opioids out of concern for regulatory oversight, addiction risk, or institutional policy. As a result, cancer patients—many of whom are opioid-tolerant and experiencing severe, refractory pain—are at risk of being undertreated.^17^ This tension between public health policy and individualized cancer care has created a therapeutic gap in the ED, where pain control may be delayed or inadequate, contributing to patient distress, unnecessary admissions, and erosion of trust in the healthcare system. Addressing this issue requires not only provider education but also clinical tools and pathways that support safe, appropriate opioid use in cancer patients.

Our study demonstrates that a BPA embedded within the EMR may impact key outcomes. After adjusting for confounders, BPA-directed care was associated with a reduction in OR of hospital admission, a modest improvement in pain, and a small but statistically significant reduction in ED length of stay. These effects were most pronounced in the most vulnerable subgroups: among patients with ECOG 4 performance status, BPA use was associated with a reduced the admission rate from 80% to 54%; among patients with stage 4 cancer, admission dropped from 68% to 57% with BPA guidance. These findings suggest that EMR-guided opioid dosing, tailored to a patient’s opioid history and disease severity, is associated with a reduction in unnecessary hospitalizations while improving symptom management in a time-sensitive environment.

Nonetheless, gaps remain. Over half of patients in our cohort were admitted, and among those with severe pain and poor performance status, pain remained largely refractory despite ED treatment. This underscores a need for more robust, multimodal cancer pain protocols in the ED—potentially including early palliative consultation, nonopioid adjuvants, and tailored discharge planning. The high burden of advanced disease coupled with the low utilization of hospice (only 4%) suggests missed opportunities for earlier goals-of-care discussions and care transitions. Grudzen et al^18^ have shown that early palliative care consultation for patients with cancer in the ED has improved quality of life without shortening lifespan. A subsequent Cochrane review further substantiated that palliative care consultation in the ED setting, particularly for advanced cancer, can substantially improve quality-of-life measures.^19^ Opioid adjuncts, including regional anesthetic blocks, although previously confined to the outpatient setting, are now finding traction in the ED setting. Bubic et al^20^ recently published an article detailing how regional pain management services can be deployed in acute care settings to provide safe, opioid-alternative and effective analgesia.^20^ Furthermore, 2 recent case series were published, including 1 from our group, demonstrating that erector spinae blocks could be used to safely and effectively treat refractory cancer-related abdominal pain in the ED setting.^21^^,^^22^ Targeted, personalized, EMR-guided analgesia, coupled with nonopioid adjuncts and early palliative care consultation have the potential to decrease unnecessary admissions for refractory cancer pain.

This study demonstrates that an EMR-based BPA may meaningfully improve the acute management of cancer-related pain in the ED. By offering individualized opioid dosing recommendations based on a patient’s home regimen and opioid tolerance, BPA-guided care was associated with reduced hospital admissions, improved pain control, and shorter ED stays, particularly among the most vulnerable patients with advanced disease or poor functional status. Additionally, sustained adherence to BPA recommendations across multiple opioid administrations was linked to more consistent analgesia, underscoring the importance of structured, protocol-driven approaches to pain management. These findings highlight the potential for EMR-integrated decision support to bridge gaps in acute oncology care, reduce variability in practice, and promote equitable pain relief. Future research should evaluate the long-term impact of such interventions on patient-reported outcomes, explore their integration with nonopioid modalities and palliative care pathways, and assess scalability across diverse emergency care settings.

## Author Contributions

CC conceived the study, designed the intervention, and provided study oversight. CC, RM, and KD supervised the conduct of the study and data collection. RM and KD managed the data, including quality control. CC, RM, KD, and AT analyzed the data. CC provided statistical support. CC and AT drafted the manuscript, and all authors contributed substantially to its revision. CC takes responsibility for the paper as a whole.

## Funding and Support

By *JACEP Open* policy, all authors are required to disclose any and all commercial, financial, and other relationships in any way related to the subject of this article as per ICMJE conflict of interest guidelines (see www.icmje.org). The authors have stated that no such relationships exist.

## Data Availability

Partial or complete datasets and data dictionary are available from date of article publication upon request to Dr Coyne at cjcoyne@health.ucsd.edu to investigators who provide an institutional review board letter of approval.

## Conflict of Interest

All authors have affirmed they have no conflicts of interest to declare.
