# Supplementary material for: Smart Dosing, Better Outcomes: An Electronic Medical Record Intervention for Cancer Pain in the Emergency Department
Source: J Am Coll Emerg Physicians Open. 2026 Jun 25;7(4):100449. doi: 10.1016/j.acepjo.2026.100449 (PMC13325251; doi:10.1016/j.acepjo.2026.100449)
Supplement: Supplementary Appendix 2 [file mmc2.docx]

| **Medication** | **MEDD 100–150** | **MEDD 150–200** | **MEDD 200–250** | **MEDD 250–300** | **MEDD 300–350** |
| --- | --- | --- | --- | --- | --- |
| **Morphine IV/IM** | 7.5 mg | 10 mg | 12.5 mg | 15 mg | 15 mg |
| **Hydromorphone IV/IM** | 1.5 mg | 1.75 mg | 2 mg | 2 mg | 2.5 mg |
| **Fentanyl IV/IM** | 50 mcg | 75 mcg | 100 mcg | 100 mcg | 100 mcg |
| **Morphine PO** | 30 mg | 45 mg | 45 mg | 45 mg | 60 mg |
| **Hydromorphone PO** | 4 mg | 6 mg | 6 mg | 6 mg | 8 mg |
| **Oxycodone PO** | 10 mg | 15 mg | 20 mg | 20 mg | 25 mg |
| **Oxycodone/Acetaminophen PO** | 10/325 mg | — | — | — | — |
| **Hydrocodone/Acetaminophen PO** | 10/325 mg | — | — | — | — |

Appendix 2: Medication table that BPA utilizes after calculating patient-specific MEDD. IV=intravenous, IM=intramuscular, PO=per os (oral), MEDD=morphine equivalent daily dose
